# The impact of the BDNF Val66Met genotype on intrusive memories following trauma exposure and in PTSD is moderated by sex and timing of trauma exposure

**DOI:** 10.1038/s41598-025-13812-8

**Published:** 2025-08-22

**Authors:** Emma Louise Nicholson, Michael Garry, Luke J. Ney, Ken Chia Ming Hsu, Daniel V. Zuj, Kim L. Felmingham

**Affiliations:** 1 Melbourne School of Psychological Sciences, University of Melbourne, Redmond Barry Building, Parkville, VIC 3010 Australia; 2 School of Psychological Sciences, University of Tasmania, Dynnyrne, Australia; 3 School of Psychology and Counselling, Faculty of Health, Queensland University of Technology, Brisbane, Australia; 4 Experimental Psychopathology Lab, Department of Psychology, Swansea University, Swansea, UK

**Keywords:** Genetics, Neuroscience, Psychology, Risk factors

## Abstract

Intrusive memories are a key symptom of Post-Traumatic Stress Disorder (PTSD). Brain Derived Neurotrophic Factor (BDNF) has been proposed as a possible mechanism influencing intrusive memories in PTSD given its role in synaptic plasticity and memory consolidation. The BDNF Val66Met polymorphism has been linked PTSD susceptibility and episodic memory disturbances however previous research outcomes have been variable, potentially due to a failure to control for important confounds such as sex, ethnicity, BMI, developmental stage and extent of previous trauma experiences. This study explored the relationship between the BDNF Val66Met genotype and emotional memory (intrusive memories and recall) in PTSD controlling for these factors in 276 participants: 53 with PTSD, 118 Trauma Exposed and 105 Controls. Key findings revealed the PTSD group experienced significantly more negative intrusions than Controls, and females more intrusions than males, however there were no group or sex differences in negative memory recall. When developmental stage of trauma was considered in a traumatised sub-sample, BDNF genotype significantly interacted with PTSD status, sex, and developmental trauma stage. This highlights the importance of controlling for sex and timing of trauma on BDNF expression in neurobiological PTSD research, however further research is needed to replicate these preliminary findings and investigate the specific epigenetic and neurobiological mechanisms involved.

**Supplementary Information:**

The online version contains supplementary material available at 10.1038/s41598-025-13812-8.

## Introduction

Emotional memory dysregulation is thought to be a core mechanism underlying Post Traumatic Stress Disorder (PTSD)^1,2^ and may lead to key symptoms such as distressing intrusive memories or flashbacks of the trauma^2^. Intrusive memories are a particularly debilitating symptom of PTSD; they are involuntary, fragmented, and associated with intense arousal^3^. Recent network analyses suggest that intrusive memories are a central symptom driving the development of other PTSD symptoms, enhancing the risk of PTSD^4^.

It is known that negative stimuli are better recalled than neutral or positive^5^, and that people with PTSD have greater negative intrusive memories than controls^6–9^. It is also well established in animal and human memory research that heightened arousal (and stress hormone release) in amygdala and hippocampal networks can strengthen the consolidation of emotional memories^10^. PTSD is associated with heightened stress hormone release at the time of trauma and dysregulation in these amygdala and hippocampal networks^11^ and these alterations are linked to intrusive memories in PTSD^9,11,12^. Therefore, the intensity of arousal in amygdala and hippocampal emotional memory networks are key mechanisms involved in intrusive memories.

Importantly, only a subset of individuals develop PTSD following trauma exposure, raising the question of what specific individual factors that influence emotional memory differ in those with PTSD? Increasing research suggests that PTSD is influenced by both genetic and environmental factors^13–15^. Brain Derived Neurotrophic Factor (BDNF) has been proposed as a possible genetic influence due to its substantial impact on memory. BDNF is recognised for its role in synaptic growth, regulation and long term potentiation (LTP) which are integral components of memory consolidation^16,17^. The action of BDNF in emotional memory networks (amygdala, hippocampus and prefrontal cortex: PFC) has been identified as being crucial for emotional memory formation, particularly in fear related memories prominent in PTSD^18,19^. In humans, the BDNF gene has a single nucleotide polymorphism (SNP) linked to the regulation of both transportation and secretion of BDNF in neurons. This genetic variation results in a substitution at codon 66, where Valine (Val) is replaced by Methionine (Met), commonly denoted as Val66Met^20^. The Met allele (Met/Met or Val/Met genotype) of the Val66Met SNP is linked to diminished activity-dependent secretion of BDNF and has been correlated with decreased episodic memory and impaired hippocampal function^21,22^. Therefore, the BDNFVal66Met SNP may be an important individual factor that moderates the intensity of intrusive memories following trauma.

A number of studies in healthy humans have examined the association between the Val66Met SNP and episodic memory where the majority found a recall memory deficit in Met carriers^21–24^. A meta-analysis examining the effects of the Val66Met SNP on declarative memory and its neural correlates found the Val66Met SNP significantly modulated memory function and the structure and physiology of the hippocampus with carriers of the Met allele being adversely affected^25^. However, the authors noted these results needed to be interpreted with caution due to the significant heterogeneity of the included studies in terms of participant demographics, differing study methods, stimuli and measurements used.

Few studies have examined emotional memory and the BDNF Val66Met SNP, with mixed results. In one healthy control study, no genotypic difference was found for immediate negative or neutral memory recall, but after 24 h, ValVal carriers had significantly better 24 h positive memory recall than Met carriers^26^. Another study found Met carriers displayed increased hippocampal activation for negative words compared to ValVal carriers^27^. An fMRI study examined the effects of the Val66Met SNP on amygdala activation to emotional stimuli^28^ and found that Met carriers had significantly stronger amygdala activation to negative stimuli compared to ValVal carriers, however no emotional memory measure was included. Further, a structural imaging study found that Val66Met carriers with prior trauma exposure had altered structural integrity in hippocampal and amygdala networks that predicted greater arousal and anxiety, but again no emotional memory measure was employed^29^. Taken together, this evidence suggests that the BDNFVal66Met SNP may influence amygdala and hippocampal networks, arousal and anxiety, which are implicated in strengthening memory consolidation, and this effect may exacerbate existing dysregulation in these processes in PTSD. Therefore, the BDNFVal66met SNP may be an important individual factor that interacts with PTSD to increase intrusive memories.

Only two previous studies have examined emotional memory and BDNF in PTSD populations however both studies examined emotional memory recognition rather than intrusive memories^30,31^. While Hori et al.^30^ found a significant negative memory bias in PTSD patients with the Met allele compared to healthy controls, in our previous study both control and PTSD groups displayed a memory deficit for Met participants^31^. Given the centrality of intrusions in PTSD, there is a need for further investigation of the effects of the BDNF Val66Met SNP on emotional memory recall and intrusions, in PTSD.

Importantly, in the broader literature on the BDNFVal66Met genotype and PTSD risk, there has been considerable variability, with reviews emphasising the need to control for sex, ethnicity, BMI and previous trauma exposure as these have all been shown to influence BDNF expression and may contribute to variability of findings^32,33^. For example, the frequency of the BDNFVal66Met SNP is much higher in Asian than Caucasian samples^33,34^, and differential relationships of BDNF Val66Met SNPs to PTSD risk have been identified^20,36–41^. Secondly, BMI has been shown to influence BDNF expression in human studies^42–47^. Additionally, previous trauma and the stage of development that trauma occurred (child or adult) are also important factors to consider. Early life stress refers to a wide range of negative and stressful experiences (e.g. abuse, neglect, separation from caregivers, loss of a parent etc.) that occur from infancy through childhood and adolescence and is a major risk factor for developing PTSD in later life^48–50^. Exposure to early life stress and chronic cortisol exposure modulates BDNF expression and induces long term changes in amygdala and hippocampal networks critical for emotional memory consolidation^51,52^. Finally, sex is also important to consider as PTSD is twice as prevalent in females than males^53,54^ and BDNF expression is robustly affected by sex steroid hormones in rodent and human research^55–61^. This is particularly the case with estradiol as it induces BDNF expression which mediates hippocampal function^61–63^.

As noted above, sex steroid hormones and exposure to chronic stress/cortisol have been shown to strongly influence BDNF expression in animal research. This provides a basis for the Stress Sensitivity Hypothesis, which argues that the timing, duration and intensity of stress and cortisol exposure interacts with the Val66Met SNP, potentially in a sex specific manner, to affect BDNF expression and memory effects^15^. Therefore, it is critical to control for sex, prior trauma exposure and its developmental timing, ethnicity and BMI when considering the effects of BDNFVal66met SNP on memory in PTSD.

In summary, there exist strong relationships between emotional memory consolidation and PTSD, and between the BDNF Val66Met SNP and episodic memory. The BDNF Val66Met genotype has also been shown to influence amygdala and hippocampal networks that are critical for memory consolidation, and these effects may be stronger in PTSD populations who have existing dysregulation in these networks. Therefore, the BDNFVal66met may be a powerful individual factor moderating emotional memory function, and this may be stronger in PTSD groups. This study aims to examine the influence of the BDNFVal66met genotype on emotional memory (intrusive memories and recall) in healthy controls, trauma-exposed and PTSD populations, whilst controlling for sex, timing of trauma exposure, ethnicity and BMI. Given that no previous studies have examined intrusive memory in relation to PTSD and the BDNF Val66Met genotype whilst controlling for confounds of sex, BMI, ethnicity or timing of trauma, this study is exploratory. Our hypothesis is therefore non directional, that is, BDNF Val66Met and PTSD will interact to influence intrusive memories and memory recall.

## Results

Demographic and clinical data for the sample are displayed in Table 1. There were no significant sex differences in the groups however Games-Howell post hoc tests revealed that the Control group was significantly younger and had lower BMI than the TE and PTSD groups with no significant differences between TE/PTSD groups for age (*p* = 0.24) or BMI (*p* = 0.29). Depression, anxiety, stress, PCL, and AUDIT scores were significantly higher in the PTSD group compared to TE and Controls (see Supplementary Table S1). The TE group had significantly higher scores for PCL (*p* < 0.001) and stress (*p* = 0.001) than Controls but there were no significant differences between TE/Controls for depression (*p* = 0.08), anxiety (*p* = 0.31) and AUDIT (*p* = 0.70) scores. Refer to Supplementary Information for full post-hoc analyses of demographic and clinical data.

**Table 1:** Mean scores (standard deviations), significance and effect sizes of demographic and clinical measures for participants in the PTSD, TE and control groups.

Measure	Control (*n* = 105)	TE (*n* = 118)	PTSD (*n* = 53)	Total (*n* = 276)	Test statistic	*P*	Effect size ɳ_p_^2^
Age	22.11(6.00)	27.17(10.48)	30.36(12.35)	25.86(9.96)	*F* = 15.29	< 0.001	0.10
DASS - dep	2.22(2.73)	3.12(3.47)	8.13(5.07)	3.74(4.19)	*F* = 50.94	< 0.001	0.27
Anxiety	2.19(2.92)	2.75(2.79)	8.11(4.32)	3.57(3.88)	*F* = 67.64	< 0.001	0.33
Stress	3.50(3.19)	5.16(3.74)	11.87(4.78)	5.82(4.80)	*F* = 92.49	< 0.001	0.40
PCL	5.48(6.21)	12.08(9.57)	40.72(14.36)	15.07(16.07)	*F* = 244.4	< 0.001	0.64
AUDIT	4.57(4.08)	5.03(4.48)	7.55(5.91)	5.34(4.77)	*F* = 7.65	< 0.001	0.05
BMI	23.01(3.38)	24.58(4.89)	26.00(5.80)	24.25(4.68)	*F* = 7.84	< 0.001	0.05
Sex	59F, 46 M	72F, 46 M	37F, 16 M	168F, 108 M	χ^2^ = 2.75	0.25	
Meds	2yes/103 no	7 yes/111 no	10 yes/43 no	19 yes/257 no	χ^2^ = 16.78	< 0.001	

*n* = number of participants in group; DASS = Depression Anxiety and Stress Scale; PCL = PTSD Checklist; AUDIT = Alcohol Use Disorder Identification Test; BMI = Body Mass Index; refer to Supplementary Information for medication type/distribution; Meds: included anti-depressants, anxiolytics, benzodiazepines and mood stabilisers (2 × meds in control group were prescribed amitriptyline for pain relief not depression)

### BDNF allele frequency distribution and demographic results

Met allele carriers (Val/Met and Met/Met genotypes) were combined (into Val/Met) due to the rarity of the Met/Met genotype in Caucasian populations which can hinder relevant analysis^64^. As displayed in Table 2, participant (*N* = 276) genotype frequencies were 58% Val/Val (*n* = 160) and 42% Val/Met (*n* = 116) which did not differ across the groups (χ^2^ = 2.74, *p* = 0.25). Distribution of the BDNF genotype was in Hardy–Weinberg equilibrium (χ^2^ = 2.24, *p* = 0.13).

**Table 2:** BDNF genotype and sex frequency distribution across groups.

Genotype	Sex	Control	TE	PTSD	Total
ValVal	Male	27	29	8	64
	Female	30	40	26	96
	Total	57	69	34	160
ValMet	Male	19	17	8	44
	Female	29	32	11	72
	Total	48	49	19	116
Total	Male	46	46	16	108
	Female	59	72	37	168
	Total	105	118	53	276

Potential differences between genotypes across groups for clinical and demographic variables were also analysed using one-way ANOVAs. Results showed significant differences between genotypes for BMI [*F*(1, 275) = 13.53, *p* < 0.001] with PTSD (*p* = 0.01) and TE (*p* = *0.0*5*)* groups having a significantly higher BMI than controls (no significant BMI differences between PTSD and TE groups), however there were no significant differences for AUDIT scores, age, depression, anxiety or stress (all *p* > 0.12). Table 3 displays BDNF Val66Met x ethnicity distribution across the groups. For ethnicity x group, chi-square tests of independence were not significant for ValVals [χ^2^ (4, *N* = 160) = 4.44, *p* = 0.35], but were significant for ValMets [χ^2^ (2, *N* = 116) = 11.88, *p* = 0.01]. For ValMet participants, the proportion of Caucasian compared to Asian ethnicity was significantly greater in the PTSD group, but opposite in the Control group which had a significantly greater proportion of Asian than Caucasian participants.

**Table 3:** BDNF genotype & ethnicity frequency distribution across groups.

Genotype	Group	Ethnicity		
		Caucasian	Asian	Total
Val/Val	Control	46	11	57
	TE	60	9	69
	PTSD	32	2	34
	Total	138	22	160
Val/Met	Control	18	30	48
	TE	25	24	49
	PTSD	16	3	19
	Total	59	57	116
Total	Control	64	41	105
	TE	85	33	118
	PTSD	48	5	53
	Total	197	79	276

### Intrusive memories

#### Number of intrusive memories across valence

**Fig. 1:**
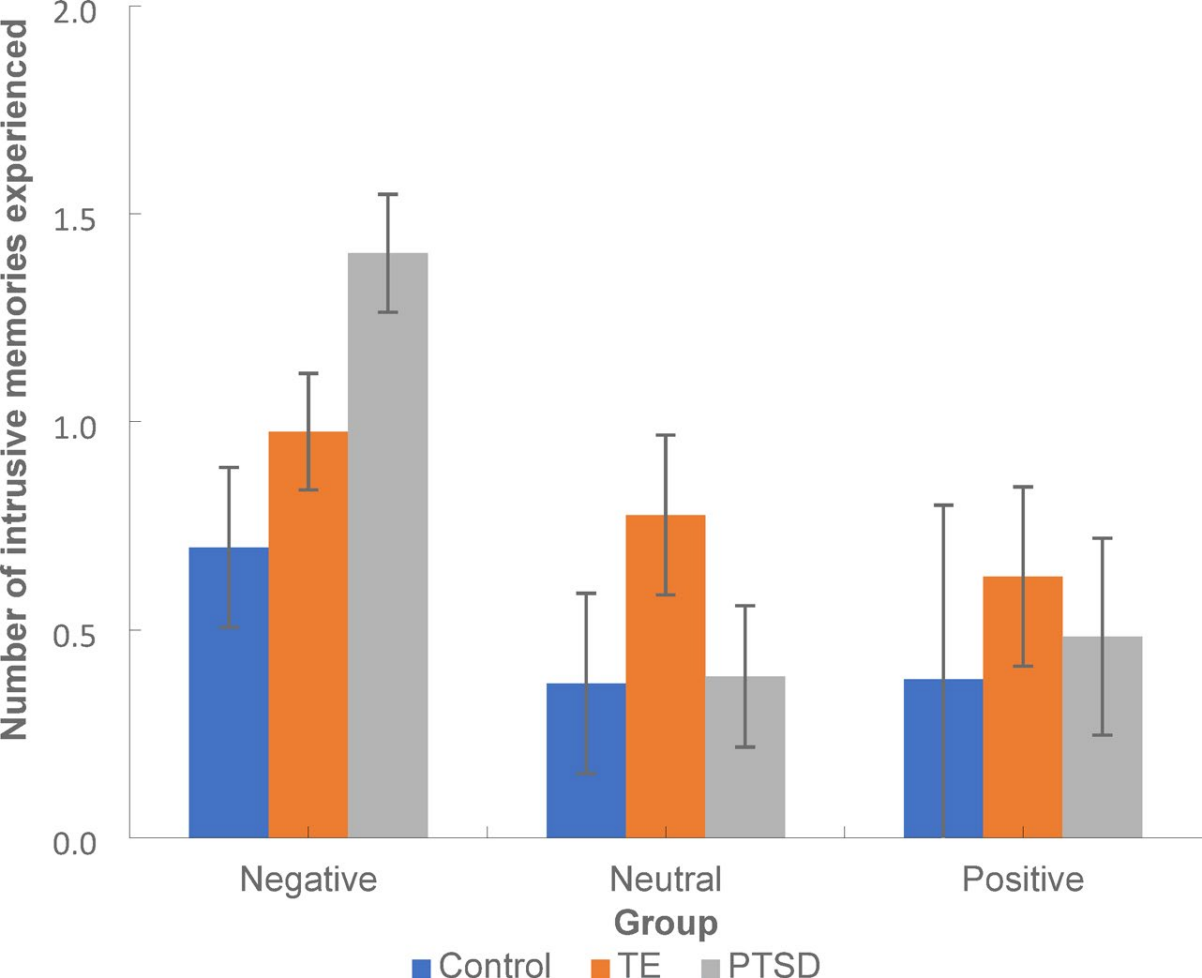
Mean number of positive, neutral and negative intrusive memories experienced across the PTSD, TE and control groups. *Note*: Error bars: 95% CI. TE = Trauma exposed but non-PTSD participants. PTSD = participants with posttraumatic stress disorder.

Intrusive memory data were initially analysed with a GLiM mixed model (negative binomial log link) using Group as a between-group factor and valence as a within-group factor. There was a significant main effect of group [*F*(2,878) = 11.68, *p* < 0.001], valence [*F*(2, 878) = 26.26, *p* < 0.001] and a significant group x valence interaction [*F*(4,878) = 3.50, *p* = 0.01]. As seen in Fig. 1, the interaction revealed the PTSD group reported significantly more negative intrusive memories than Controls (*p* = 0.002, 95% CI [0.22, 1.20) however there were no significant differences between PTSD and TE groups (*p* = 0.07, 95% CI [− 0.03, 0.89]) or TE and Controls (*p* = 0.07, 95% CI [− 0.02, 0.57]) in the number of negative intrusive memories experienced. For neutral intrusive memories, the TE group experienced significantly more than either the PTSD (*p* = 0.003, 95% CI [0.11, 0.67) or Control groups (*p* < 0.001, 95% CI [0.15, 0.66), but there were no significant differences between PTSD and Controls (*p* = 0.88, 95% CI [− 0.19, 0.23]). With positive intrusive memories, the TE group again had significantly more intrusive memories than Controls (*p* = 0.002, 95% CI [0.22, 1.20) with no significant differences between the TE and PTSD groups (*p* = 0.44, 95% CI [− 0.42, 0.14]).

The predominant effects were found in negative intrusive memories, with relative floor effects in neutral and positive intrusive memories. Therefore negative intrusive memories were the focus of subsequent analyses. These were investigated using a GLiM (negative binomial log link). This model included the number of negative intrusive memories experienced as the response variable, with predictors of group, BDNF genotype, sex and covariates of BMI and ethnicity. The analyses revealed significant main effects of group and sex (ref. Table 4) however there were no other significant main or interaction effects (all *p* > 0.19).

**Table 4:** Significant main and interaction effects for intrusive memories (IMs) reported across all groups and with developmental trauma included as a factor in TE and PTSD groups.

Predictor	Significant Effect	N	*df*	χ^2^	*p*
IMs	Group (CTL, TE, PTSD)	276	2	29.30	< 0.001
	Sex (CTL, TE, PTSD)	276	2	17.57	< 0.001
IMs reported in TE/PTSD only when developmental trauma stage included					
	Group	165	1	12.79	< 0.001
	Genotype	165	1	6.70	0.010
	Sex	165	1	22.46	< 0.001
	Group x Genotype	165	1	6.27	0.012
	Group x Child/Adult	165	1	4.76	0.029
	Group x Genotype x Sex	165	1	4.87	0.027
	Genotype x Child/Adult x Sex	165	1	10.33	0.001

The significant main effects showed PTSD experienced significantly more negative intrusive memories than TE (*p* = 0.011, 95% CI [0.18, 1.03]) and Control groups (*p* < 0.001, 95% CI [0.47, 1.44]), with TE also experiencing significantly more intrusive memories than Controls (*p* = 0.002, 95% CI [0.11, 0.59]). Females experienced significantly more intrusive memories than males (*p* < 001, 95% CI [0.32, 0.77]). Please refer to Supplementary information (Tables S2a-d) for intrusive memory means).

#### Sub-analysis: number of traumas and developmental stage traumas were experienced

**Fig. 2:**
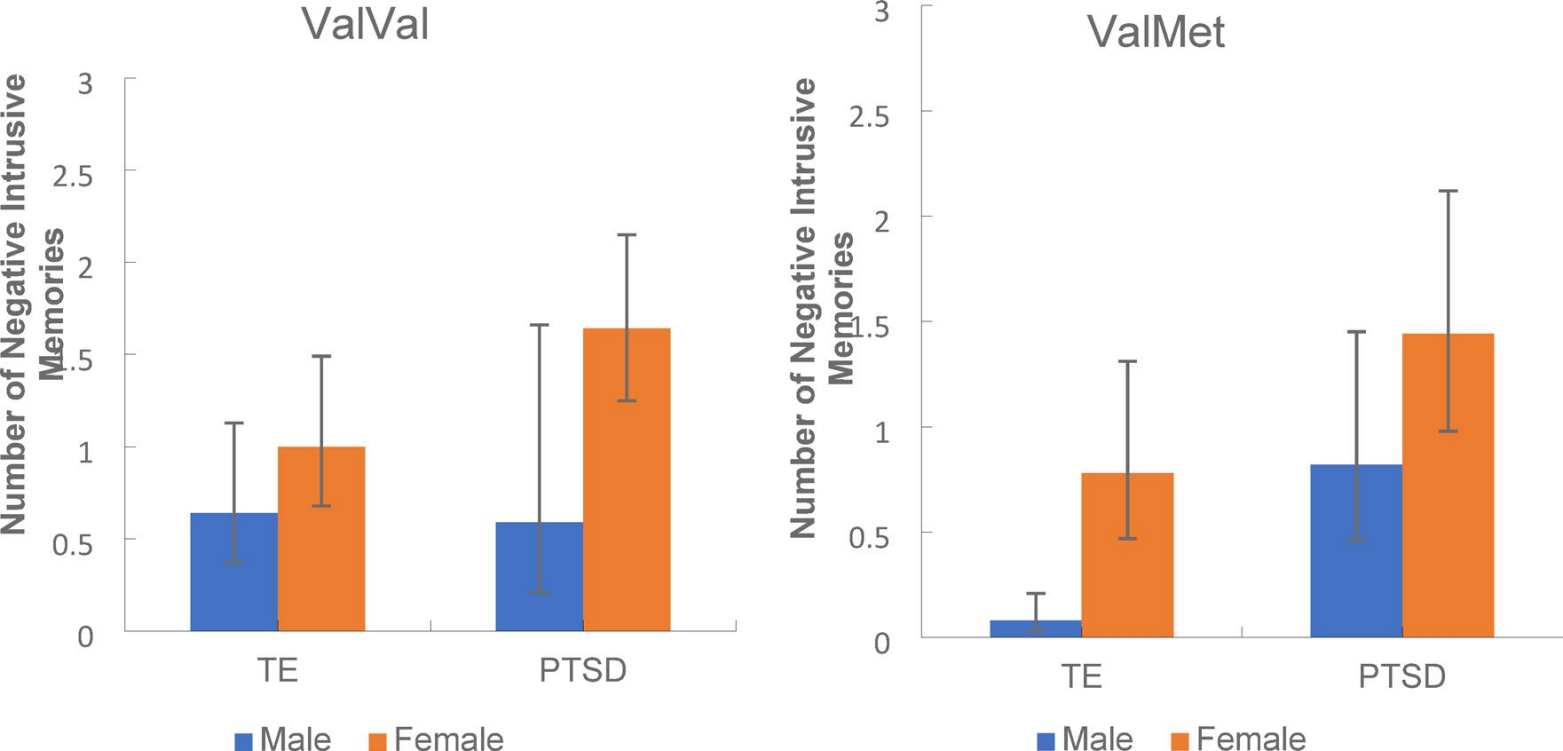
Mean number of negative intrusive memories experienced for males and females with the ValVal (**a**) and ValMet (**b**) genotypes in TE and PTSD groups when developmental stage (child/adult) of trauma was added as an additional factor. *Note*: Error bars: 95% CI.

The number of traumas experienced and developmental stage of trauma (child or adult) were added separately to the previous GLiM (negative binomial) model for the TE and PTSD groups. This resulted in several significant main and interaction effects as reported above. Figure 2a and b show the group x genotype x sex interaction when developmental stage of trauma was added as a factor to the original GLiM model.

This 3-way interaction revealed there was a significant group x sex interaction for ValVal but not in ValMet participants. For ValMet participants, although females experienced more intrusive memories than males and PTSD experienced more intrusive memories than the TE group, there was no significant group x sex interaction. However in ValVal participants, there was a significant group x sex interaction where PTSD females reported more intrusive memories than TE females, however for males, there was no such PTSD effect; there were no differences between the PTSD and TE groups in the number of intrusive memories experienced.

**Fig. 3:**
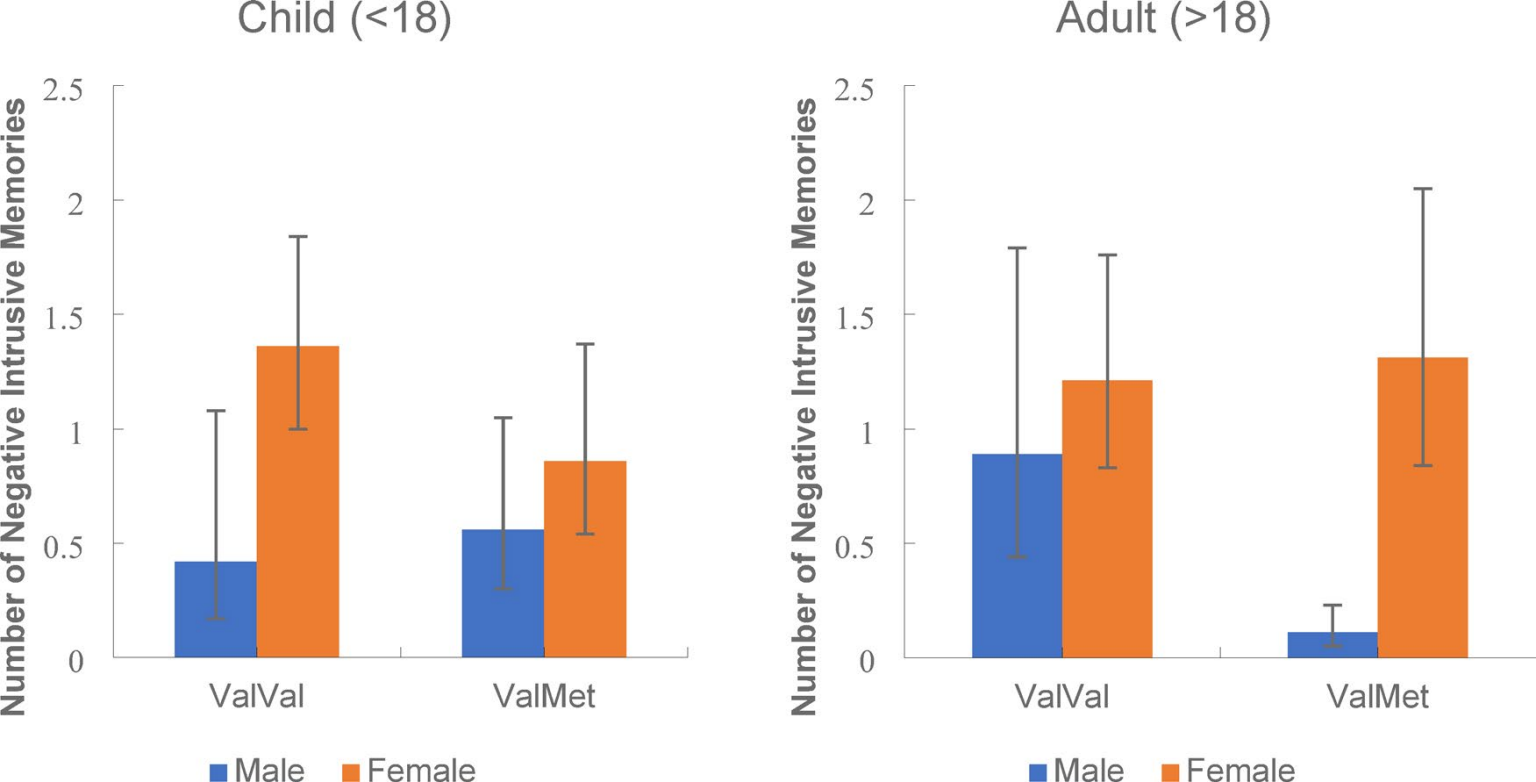
Mean number of negative intrusive memories experienced for Males and Females with the ValVal and ValMet genotype for participants who first experienced trauma either as a child (**a**) or as an adult (**b**). *Note*: Error bars: 95% CI.

The second 3-way interaction of genotype x sex x developmental trauma stage is illustrated in Fig. 3a and b.

For this 3-way interaction, the sex x genotype interaction for those who first experienced trauma as a child was significantly different to those who experienced trauma as an adult. Exploring this further, although there was an overall significant main effect of sex where females had significantly more intrusive memories than males (*p* < 0.001, 95% CI [0.49, 1.06]), if trauma was first experienced in childhood, there was a significant sex x genotype interaction where females with the ValVal allele reported significantly more intrusive memories than males (*p* = 0.001, 95% CI [0.36, 1.51]) but there were no significant sex differences for ValMets. However, if trauma first occurred as an adult, there was an opposite effect, with sex differences occurring in ValMets, namely that female ValMets experienced significantly more intrusive memories than male ValMets (*p* < 0.001, 95% CI [0.62, 1.78]) while there was no significant sex difference in the ValVal adults.

When the number of traumas experienced was added as a covariate (in addition to ethnicity and BMI) to the initial GLiM analysis in the TE and PTSD groups, there were no significant interaction effects however there were the same significant main effects as the base model of group [χ^2^ (1,* N* = 171) = 9.79, *p* = 0.01] and sex [χ^2^ (1,* N* = 171) = 9.53, *p* = 0.01]. Please refer to Supplementary Information (Tables S3a-d) for intrusive memory means in the trauma exposed groups only).

#### Distress and vividness of intrusive memories

Participants rated their levels of distress and vividness of the intrusive memories experienced which were then added as separate response variables to the original GLiM model. Significant outcomes are listed in Table 5.

**Table 5:** Significant main and interaction effects for distress and vividness.

Predictor	Significant Effect	N	*df*	χ^2^	*p*
Distress	Group (CTL, TE, PTSD)	185	2	49.55	< 0.001
Vividness	Group (CTL, TE, PTSD	183	2	18.66	< 0.001
Vividness in TE/PTSD when Developmental Stage included	Group	124	1	14.18	< 0.001
	Genotype	124	1	7.03	0.001
	Sex	124	1	6.08	0.014
	Child/Adult	124	1	4.09	0.043
	Group x Child/Adult	124	1	6.21	0.013
	Genotype x Child/Adult x Sex	124	1	6.07	0.011

CTL = Control; TE = Trauma-exposed but non-PTSD; PTSD = posttraumatic stress disorder

For levels of distress, there was a main effect of group such that PTSD were significantly more distressed by the intrusive memories than the TE (*p* < 0.001, 95% CI [0.72, 1.58] and Control groups (*p* < 0.001, 95% CI [0.83, 1.08] but no significant differences occurred between the TE and Control groups. There were no other significant main or interaction effects (all > 0.07). The addition of developmental trauma stage and number of traumas to the distress model had no significant effects.

For vividness of intrusive memories, there was again a main effect of group where intrusive memories experienced by the PTSD group were significantly more vivid than for those in the TE (*p* = 0.001, 95% CI [0.26, 1.49] and Control groups (*p* < 0.001, 95% CI [0.59, 1.89], with no significant differences between TE and Controls (*p* = 0.18, 95% CI [− 0.16, 0.88]. There were no other significant main or interaction effects. Adding the developmental trauma stage to the original GLiM Vividness model resulted in several significant main and interaction effects as per Table 5.

**Fig. 4:**
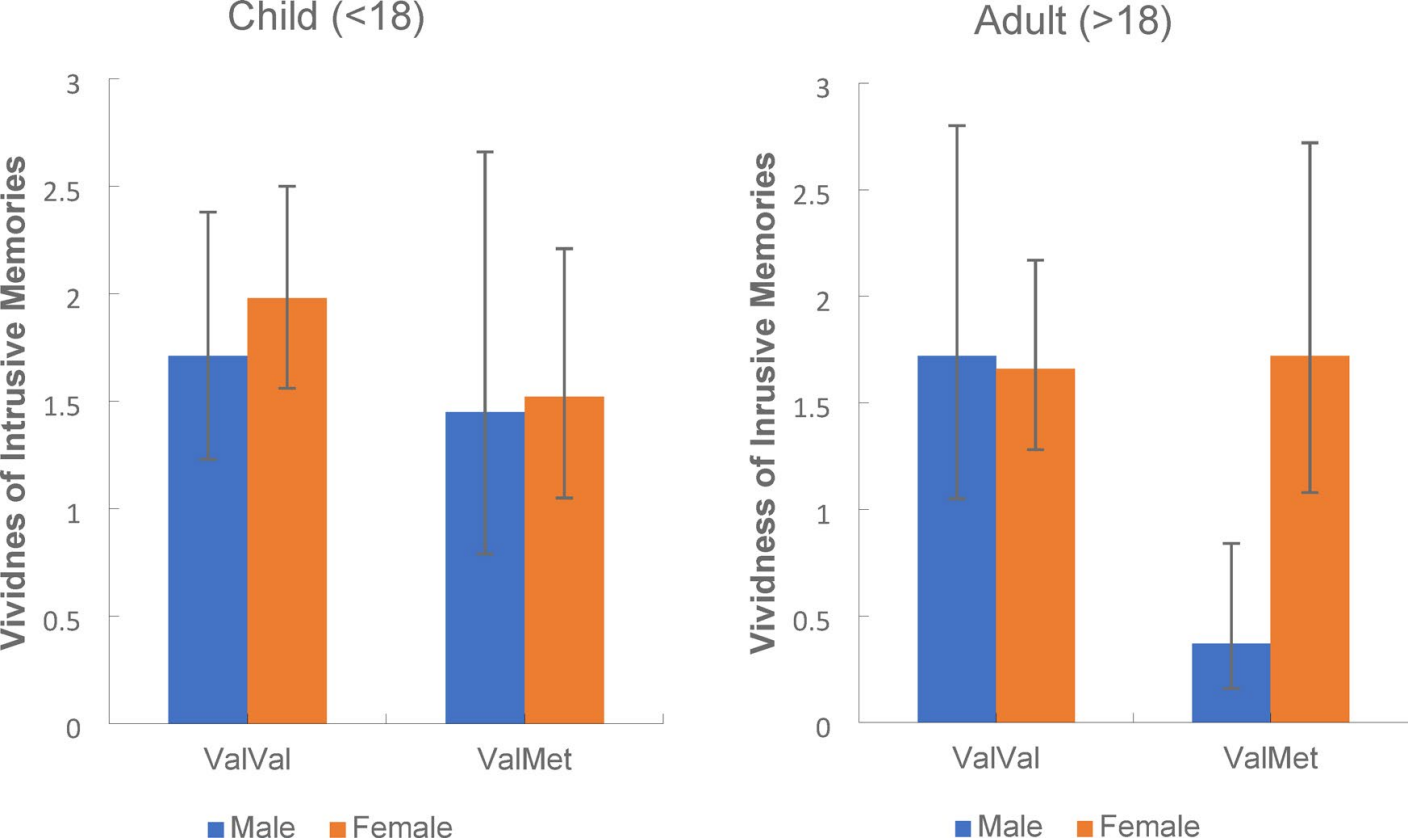
Mean vividness rating of intrusive memories experienced for Males and Females with the ValVal and ValMet genotype for participants who first experienced trauma either as a child (**a**) or as an adult (**b**). *Note*: Error bars: 95% CI.

As displayed in Fig. 4a and b depicting the genotype x sex x developmental trauma stage interaction, the sex x genotype interaction for those who first experienced trauma as a child was significantly different from those who experienced trauma as an adult. If trauma was first experienced in childhood, there was no significant sex x genotype interaction (*p* = 1.0, 95% CI [− 0.86, 1.39]). However when trauma first occurred as an adult, there was a significant interaction between sex and genotype such that intrusive memories were significantly more vivid for ValMet females than males (*p* = 0.03, 95% CI [0.07, 2.64]) although there were no significant sex differences in ValVals (*p* = 1.0, 95% CI [− 1.23, 1.34]). When the number of traumas experienced was added as a covariate to the original Vividness GLiM, there was a main effect of group [χ^2^ (1,* N* = 128) = 8.68, *p* = 0.001] but no other main or interaction effects (all *p* > 0.11). Please refer to Supplementary information (Tables S4a-d, S5a-d and S6a-d)for distress and vividness means.

### Memory recall

#### Number of memories correctly recalled across valence

**Fig. 5:**
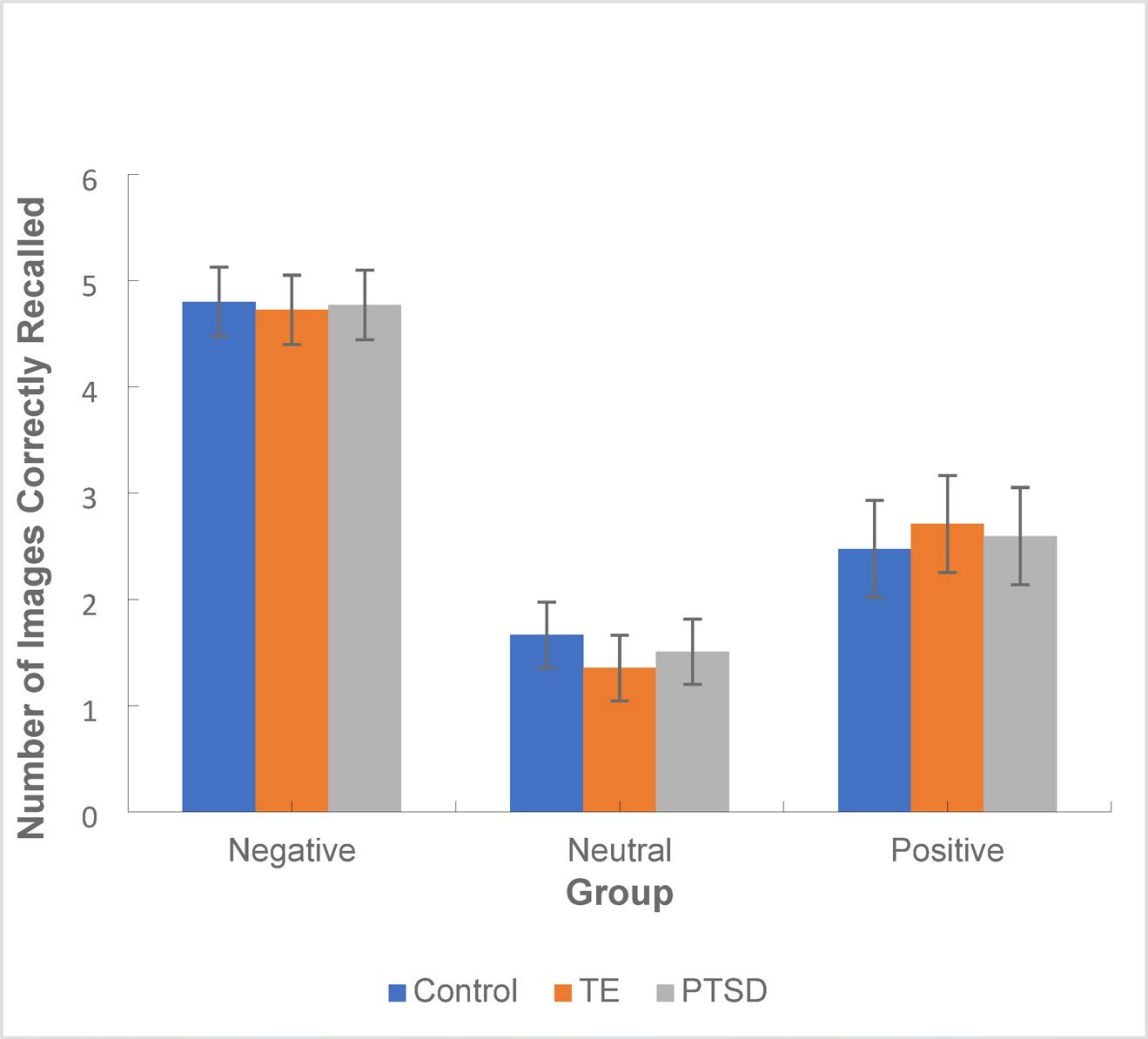
Mean number of positive, neutral and negative images correctly recalled across the PTSD, TE and control groups. *Note*: Error bars: 95% CI.

Delayed memory recall was analysed with a GLiM mixed linear model including a between subject factor of group and within factor of valence. Figure 5 displays the mean number of positive, neutral and negative memories correctly recalled across the groups.

There was no significant main effect of group [*F*(2,867) = 0.05, *p* = 0.95] but there was a significant main effect of valence [*F*(2,867) = 336.71, *p* < 0.001] which showed negative images were recalled significantly more than positive (*p* < 0.001, 95% CI [1.87, 2.46]) or neutral images (*p* < 0.001, 95% CI [2.95, 3.56]) and significantly more positive images were recalled than neutral images (*p* < 0.001, 95% CI [0.83,1.34]). There was no significant interaction effect between group and valence [*F*(4,867) = 1.03, *p* = 0.39].

#### Negative memory recall

A GLiM (normal distribution, identity link) was then used to further analyse negative recall. The model included the number of negative images correctly recalled as the dependent variable with predictors of BDNF genotype, group and sex along with covariates of ethnicity and BMI. This model revealed no significant main effects of group [χ^2^ (2,* N* = 276) = 0.15, *p* = 0.93], genotype [χ^2^ (1, *N* = 276) = 0.75, *p* = 0.39] or sex [χ^2^ (1,* N* = 276) = 0.73, *p* = 0.39] and no significant interaction effects (all *p* > 0.08). Adding factors of age, DASS or AUDIT scores to the previous models also did not result in any significant effects.

#### Sub-analysis: number of traumas experienced and stage of life trauma occurred

A sub-analysis was conducted in the groups previously exposed to trauma (PTSD and TE) to investigate the effects of cumulative trauma (the number of traumas experienced) and timing of trauma (whether trauma was experienced as a child or adult). These factors were added in separate models as additional factors to the previous GLiM model. Adding developmental stage of trauma (child/adult) to the model and cumulative trauma in a second model resulted in no significant main or interaction effects for either factor (all *p* > 0.14 and *p* > 0.06 respectively). Please refer to Supplementary Information (Tables S7-S8) for memory recall means.

## Discussion

This study examined the relationship between the BDNF Val66Met genotype and emotional memory consolidation (reflected in delayed memory recall and intrusive memories) in Control, TE and PTSD groups. Key findings revealed greater intrusions and recalled memories for negative images relative to neutral images, confirming the well known negative bias effect of emotional memory^5–7^. In addition, there were significantly greater numbers of negative intrusive memories in the PTSD group compared to controls, and in females rather than males. These patterns of findings accord with previous literature^9,65–70^. Participants with PTSD also reported intrusive memories as more distressing and vivid than other groups, in line with previous findings^3,4^. There were no significant effects of group or sex on memory recall, which accords with some previous empirical research^9,71,72^ and suggests the PTSD negative memory bias is stronger in intrusive memories than recall.

Our key prediction, that the BDNF Val66Met genotype would interact with PTSD group status to influence emotional memory, was not confirmed in the overall analysis. There were no significant effects of BDNF genotype on intrusive memory or recall, or interactions between the BDNF Val66Met genotype and group on memory measures in the whole sample. This null effect may have resulted from variability in the data from uncontrolled confounds. Research findings examining emotional memory and the BDNF Val66Met SNP have been mixed^26,27^ with several review papers on BDNF, memory and PTSD noting that the literature is `consistently inconsistent’ and that several confounding variables may account for this inconsistency by influencing BDNF expression such as ethnicity, sex, estrogen^32,33,63^, and more recently, developmental stage of trauma^40^. Given these criticisms, we explored whether BDNF genotype interacted with PTSD to influence emotional memory while controlling for timing of trauma, sex, BMI and ethnicity in a sub-sample comprising our TE and PTSD groups. No significant interaction effects were found when the number of traumas experienced was added as a covariate. However, when developmental stage of trauma (child or adult) was examined in a trauma exposed sub-sample, significant interactions were found between genotype, sex and group as well as genotype, sex and developmental trauma stage.

In the genotype x sex x group interaction, for the ValMet participants, results accord with previously found patterns of greater intrusive memories in PTSD patients^6–9,65–68^ and females^69,70^ but there was no group x sex interaction. In contrast, there was a significant group x sex interaction for the ValVal participants where females with PTSD reported significantly more negative intrusive memories than TE females, whereas there was no significant PTSD effect in ValVal Males. Notaras and van den Buuse^15^ in their Stress-Sensitivity Hypothesis postulated that the low expression of BDNF in the ValMet genotype coupled with glucocorticoid sensitivity from trauma exposure sensitises threat pathways and arousal networks from the limbic system and the amygdala. Therefore, the heightened stress sensitivity associated with PTSD combined with low expression of BDNF may enhance negative memory consolidation leading to increased negative intrusive memories which could explain the greater negative intrusive memories reported. The higher expression of BDNF in ValVal males could be ameliorating this stress sensitivity in our study which may be protective against the effect of PTSD. This was not found for ValVal females which may be related to the added risk element of fluctuations in estradiol in females across the menstrual cycle^15,73^ as estradiol increases BDNF expression moderating hippocampal function which is crucial for memory consolidation^61–63^. Therefore, low levels of estradiol in women during the early follicular phase of their menstrual cycle may lessen the expression of BDNF in ValVal females, leading to less protective influences of BDNF against PTSD stress sensitivity.

A significant genotype x sex x developmental trauma stage interaction was also evident in the trauma exposed participant sub-analysis. This interaction revealed that for those who experienced childhood trauma, there were sex differences in the ValVal genotype where females experienced more intrusive memories than males but not in the ValMet group. However for those with adult trauma, the effect was the opposite; sex differences were only present in the ValMet genotype where females experienced more intrusive memories than males. This pattern of findings might again relate to estradiol fluctuations and subsequent impact on BDNF expression. Estradiol fluctuations in females only occur following puberty once menses has commenced, leading to a potential synergistic effect of low BDNF expression (in the ValMet genotype) and low estradiol further restricting BDNF expression in adult females experiencing estradiol fluctuation with their menstrual cycle. This pattern was not observed in participants with childhood trauma.

Our finding of greater intrusive memories for ValVal females than males with childhood trauma align with recent research by Jin et al.^40^ who found ValVal participants with higher scores on a childhood trauma questionnaire had higher PTSD symptom severity compared to ValMet participants in a Korean population. The authors noted that these results may have been influenced by ethnicity differences as the prevalence of the Met allele is significantly higher in Asian than Caucasian populations^35^ with some studies suggesting the Val allele may be a particular risk in Asian populations^33,39,63^. Our study participants were of mixed ethnicity (Caucasian/Asian) and these findings require further investigation and replication in larger studies that can model the effects of ethnicity and sex differences in well-powered designs.

Taken together, these findings highlight the importance of controlling for important confounds that influence BDNF expression such as the developmental timing of trauma and. sex. Although the ValVal genotype is considered protective against stress due to higher BDNF expression^15^, epigenetic mechanisms which may involve the action of BDNF and exert greater impact during developmental periods can mediate differences in adult PTSD depending on previous exposure to childhood trauma^48,74^. Additionally, exposure to stress and chronic cortisol significantly reduces BDNF expression which is enduring^75,76^. These influences may negate the protective effect of the ValVal genotype which could be a contributing factor in our results. Nevertheless, our results suggest that chronic trauma exposure (with the potential involvement of chronic cortisol exposure as proposed by the Stress Sensitivity Hypothesis) are influencing BDNF expression. Furthermore, there appears to be sex specificity in the effects of the BDNF Val66Met SNP, which may relate to the regulation of BDNF expression by estradiol via the inclusion of estrogen response elements in the BDNF Val66Met genotype^15^.

Future research needs to examine this further in well powered studies, including stress and sex steroid hormonal measures along with well characterised trauma histories covering not just the developmental timing of trauma, but the timing of trauma exposure around puberty, the interaction of sex and stress hormones, and also cumulative trauma. This is particularly relevant for individuals in occupations involving exposure to repetitive trauma such as first-responders or the military. Future large-scale research studies that can model more precise interactions and neurobiological mechanisms may eventually lead to a platform of knowledge to inform sex-specific, gene-based and personalised interventions.

## Limitations

Whilst novel, our study was exploratory and our findings need to be qualified by some limitations. A main limitation was the relatively modest sample size to explore our moderation analyses which makes our findings preliminary and in need of replication. Whilst previous literature highlighted the need to examine confounds of sex, ethnicity, BMI and prior trauma, our study was likely underpowered to model interactive effects, therefore there was a risk of type 2 errors. Accordingly, these findings need to be replicated in much larger samples that are well powered to model these moderating effects. This is likely to be beyond the scope of a single mechanistic study if examining clinical populations, so future research will require multi-site studies with harmonised data collection. Ideally, sample sizes should be large enough to enable adequate representation of the Met genotype. Due to the limited number of participants with the MetMet genotype, MetMet and ValMet participants were combined for comparison against ValVal participants which is standard practice^64^ but may have reduced the strength of any genotypic effect. This will enable greater translational comparison with animal research^19^.

A further limitation of the current study was that we examined a single candidate genotype, the BDNF Val66Met. With the growing recognition of polygenic influences, there is an increasing emphasis (with the PTSD Consortium) on GWAS studies to identify multiple key genetic influences underlying PTSD^77^. However, a limitation with current GWAS datasets is the lack of deep clinical, mechanistic and phenotypic analyses which can address hypothesis-driven questions. Future research should be powered to examine multiple key genotypes and their interactions on emotional memory mechanisms in PTSD, and examine PTSD symptom clusters alongside total symptom scores, and the more recent focus on complex PTSD, for deeper insights and phenotyping.

A final and important limitation was the use of cross-sectional data and moderation analyses. We selected a moderation analysis as we were testing the impact of individual factors (BDNF Val66Met genotype, sex, timing of trauma exposure) and how they exacerbated existing emotional memory dysregulation in PTSD. However, an optimal design to examine more causal mechanisms underpinning PTSD and to test theoretical models of how PTSD develops would be to employ longitudinal data in mediation models.

## Conclusion

To our knowledge this is the first study to examine the influence of the BDNF Val66Met genotype and emotional memory consolidation (reflected in delayed memory recall and intrusive memories) in PTSD, TE and healthy control groups. Key findings revealed greater negative intrusions in the PTSD group and in females, but no significant findings for memory recall. There were no overall significant effects of the BDNF Val66Met genotype on intrusive memories, but in the traumatised subsample, BDNF genotype interacted with sex, group and developmental trauma. Specifically, the high expression (ValVal) BDNF genotype appeared protective against PTSD effects on intrusive memories in males but not females, and females with adult trauma exposure and the low-expression BDNF (Met) genotype had greater negative intrusions than females with childhood trauma and the Met genotype. Both these effects may be explained, at least partially, by fluctuations in estradiol across the menstrual cycle, which is known to influence BDNF expression. This highlights the importance of controlling for neurobiological influences on BDNF expression such as ethnicity, sex, estradiol and developmental stage of trauma. However further well-powered studies are required to replicate these findings and to investigate the specific epigenetic and neurobiological processes involved.

## Method

### Participants

Testing was held on two separate sites at the Universities of Melbourne and Tasmania. The study included 276 participants as a subset from a total sample of 307 as part of a broader emotional memory study which also included a separate recognition memory component^31^. The participants (168 females, 108 males; age *M* = 25.9) consisted of both university students and local community members recruited through advertisements placed in private psychology clinics, community noticeboards and both universities. Participants received either $100 or course credit for students. The study was approved by both the Tasmanian and Victorian Medical Human Research Ethics Committees. All experiments were performed in accordance with relevant guidelines and regulations.

The participants were allocated to one of three groups based on their previous exposure to trauma. The control group consisted of 105 participants (59 female, 46 male, age *M* = 22.11, *SD* = 6.0) who reported no exposure to a Criterion A traumatic event and very few symptoms on the PTSD Checklist (PCL5)^78^. The Trauma-exposed (TE) group (*N* = 118, 72 female, 46 male, age *M* = 27.17 *SD* = 10.48) were classified as participants who had previously experienced a Criterion A traumatic event identified by the Traumatic Events Questionnaire (TEQ)^79^ but who reported a total score < 25 and no re-experiencing symptoms. Those that had experienced a Criterion A traumatic event and reported consistent symptoms with DSM-5^80^ diagnostic criteria for PTSD on the PCL^78^ with a PCL total score > 33 were classified as PTSD (*N* = 53, Female = 37, Male = 16, age *M* = 30.36, *SD* = 12.35). The PTSD group were a civilian sample (including 7 ex-service personnel) with a range of traumas experienced including sexual or interpersonal assault, combat/war zone experience, natural disasters and motor vehicle accidents. Participants were all below age 65 to control for potential memory confounds. Exclusions included pregnancy, breast feeding, reported serious medical illness, psychosis, suicidality, substance dependence, neurological damage or head injury.

### Materials and measures

*International affective picture system images*^81^. Twenty emotionally negative images (mean valence: 2.30, arousal: 6.18), 20 neutral images (mean valence: 4.99, arousal: 2.75) and 20 positive images (mean valence: 7.49, arousal: 4.42) were selected using normative data and stimuli from the IAPS^62^ and displayed on a computer screen.

*Depression, anxiety and stress scale*^82^. The DASS is a 42 item self-report questionnaire which assesses the severity of current depressed, anxious and stress mood states. Each item is rated on a four-point Likert Scale of severity or frequency of the participants’ experiences over the last week. Scores range from 0 (the item “did not apply at all”) to 3 (“applies very much, or most of the time”). The scales have a reliability score (Cronbach’s Alpha) of 0.84 for Anxiety, 0.90 for Stress and 0.91 for the Depression scales in the normative sample. This scale was used to index levels of depressed, anxious and stressed mood during the week of testing.

*PCL*. The PCL was used to screen for PTSD symptomatology. The PCL-5^78^ was used at the Melbourne testing site while an earlier version (PCL-IV)^83^ was used in Hobart. The PCL-5 is a standardized 20-item self-report measure of PTSD symptomatology corresponding to the DSM-5 symptom criteria for PTSD^80^. Respondents indicate how much they have been affected by each symptom in the past month using five-point Likert scales with scores ranging from 1 “Not at all” to 5 “Extremely.” The PCL provides an ordinal measure of the severity of PTSD symptoms and suggests a cut-off of 31–33 for probable PTSD^78^. A symptom is scored as “moderate” is considered a symptom endorsement, and diagnostic status can be obtained by examining a pattern of endorsement of the 20 PTSD symptoms as per DSM-5^80^. PCL-5 scores range from 0 to 80. The PCL-IV^83^ is a 17 item self-report measure that mapped onto DSM-IV-TR PTSD symptom criteria^84^. Scores range from 17 to 85. Due to the fact the PCL-IV and PCL-5 were used at different testing sites, a validated procedure was utilised (PCL `Crosswalk’) to convert PCL-IV to PCL-5 scores as outlined in Moshier et al.^85^.

*TEQ (Vrana & Lauterbach, 1994)*^79^. A scale consisting of 11 dichotomous (yes/no) items regarding whether respondents have experienced a Category A trauma^80^ as well as providing the type of traumatic event experienced listing 11 Criterion A traumatic events. The TEQ was used to screen for trauma exposure.

*Alcohol Use Identification Test (AUDIT)*^86^. The AUDIT is a 10-item questionnaire covering the domains of alcohol consumption, drinking behaviour and alcohol related problems. Responses are scored on a scale of 0–4 with a maximum score of 40. Harmful is determined by a score of 8 or more and alcohol dependence a score of 16 or above. The AUDIT was used to gauge the frequency/severity of participant’s alcohol use as harmful use can negatively impact memory function.

*BMI*. BMI was recorded for each participant as obesity is a known confound and can influence BDNF expression^87^.

### Procedure

**Fig. 6:**
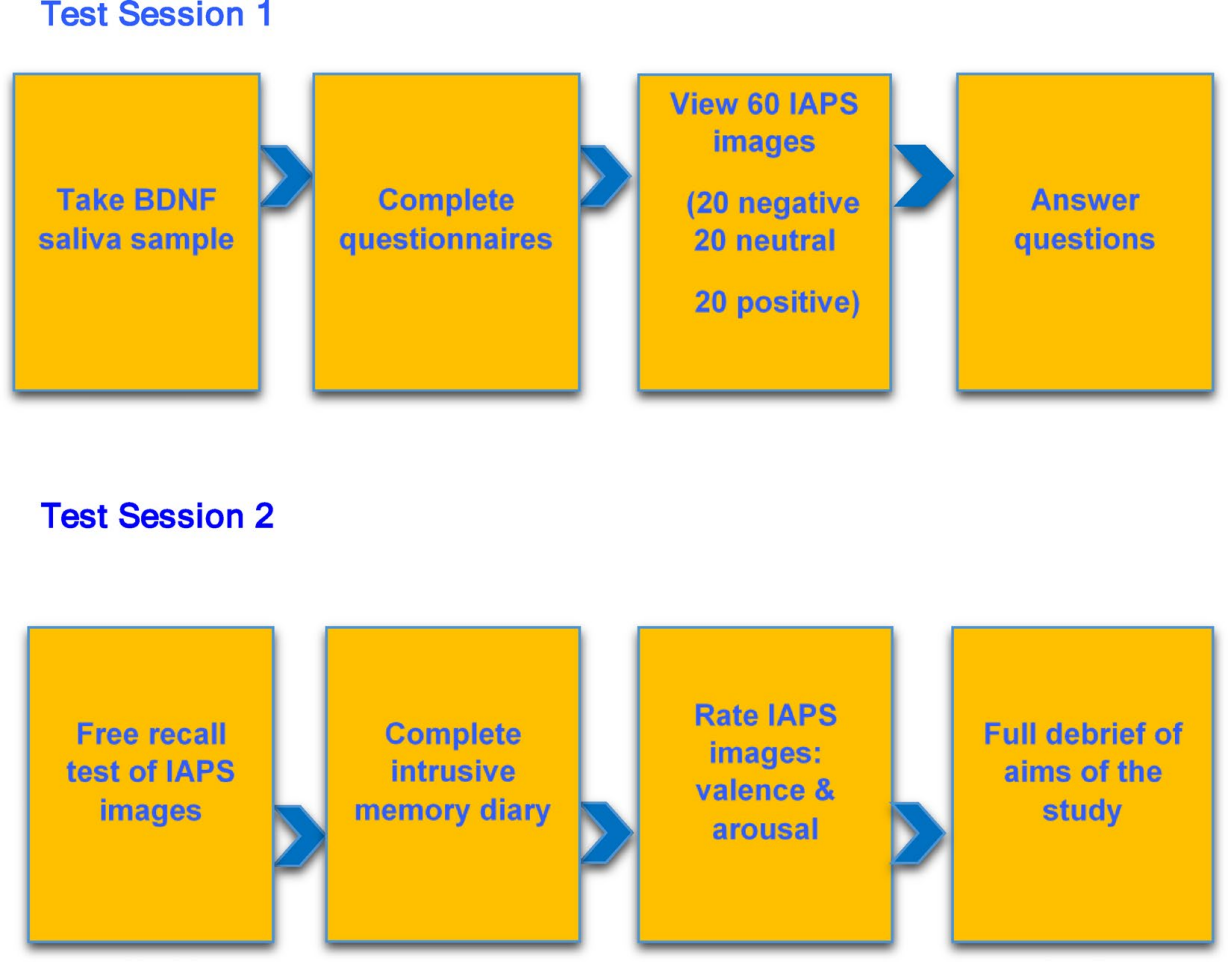
Experimental timeline.

Participants completed two testing sessions with a two-day hiatus between sessions. In session one, participants gave informed consent and were habituated to the test environment for 10 min. A BDNF saliva sample was extracted for genotyping for the BDNF Val66Met polymorphism and participants completed required questionnaires. Participants then viewed a series of images on the computer screen in front of them. Sixty IAPS images were presented, 20 negative (rated as highly unpleasant and arousing according to IAPS norms^81^), 20 neutral and 20 positive (pleasant but not arousing). Images were shown for six seconds each in block format (20 positive, 20 negative, 20 neutral) in randomised order across participants. After this, participants were instructed to return in two days to undertake similar testing procedures. To avoid priming or image rehearsal, participants were not informed they would complete a memory test at any stage. Figure 6 outlines a timeline of the procedure of the study.

At the second testing session, participants were first given a surprise memory recall task. They were instructed to write a description of as many of the IAPS images they could recall from the first test session (as per procedure described^88^). They then completed an intrusive memory diary (adapted from^89^) where they reported the content and number of intrusive memories of the IAPS images experienced (if any) during the intervening two days. Standardised prompts (e.g. Can you tell me anything else you remember about the image? Can you recall any colours in the image? Can you recall any other objects in the image? Was it day or night?) were used to report specific details of each image enabling raters to gauge the concordance of these reports against the actual IAPS images. As per procedures^88^, these reports were classified by two independent raters according to the IAPS images they represented, and an estimate of inter-rater reliability was calculated for each image. If there was disagreement between raters, that image was removed from the analysis. In total, 98% inter-rater reliability was achieved. Each IAPS image was then rated by participants on valence and arousal following standardised procedures^62^. A full debrief concerning the aims of the research was then given.

### Design and analysis

*Salivary Genomic Analysis*. Participants provided a 2 mL saliva sample using Oragene DNA self-collection kits (DNA Genotek Inc, 2012). Purification and extraction of DNA from saliva samples was completed at the Australian Genome Research Facility (AGRF). The BDNF Val66Met polymorphism was identified using an established polymerase chain reaction (PCR) method^90^. PCR amplifications were conducted using a ten µL reaction volume containing approximately 50 mg of genomic DNA. PCR amplicons were resolved on a 2% agarose gel. Genotyping was repeated to ensure accuracy, with the proportion of concordance > 99%.

*Statistical Analysis*. SPSS28 for Windows was used for all analyses. Demographic and clinical data were analysed with univariate analyses of variance (ANOVA) with Greenhouse Geiser corrections applied if required. Genotype and sex distribution were analysed using 3 × 2 chi-square tests of independence. A chi-square goodness of fit test was used to compare observed genotype frequencies with the expected genotype frequencies for an Australian population. This was to test the sample against Hardy–Weinberg equilibrium to confirm that the distribution of genotype alleles matched that of the general population^91^.

Mixed linear models were utilised to compare differences between recall and intrusive memory valence measures (positive, neutral and negative) across the three groups. For negative memory recall, as the data was normally distributed, a normal distribution (log link) model was used with the number of negative images recalled as the response variable and predictor variables of group, genotype and sex with ethnicity and BMI as covariates. For intrusive memory, due to highly skewed data that was low frequency count, a GLiM (negative binomial) analysis was used with the number of negative intrusive memories experienced as the response variable. Predictor and covariate variables were the same as the GLiM negative recall model. Two further separate negative binomial GLiM models (predictor and covariates as per previous models) were used to analyse participant’s levels of distress on viewing the images and their rating of the perceived vividness of the images. Additionally, for participants that had experienced trauma, the developmental stage in which the trauma was experienced (child or adult) was added as a factor subsequently to each model for all response measures (intrusive memory, distress, vividness, recall). Then in a separate GLiM for each response variable, the number of traumas experienced was added as a covariate. Sequential Sidak post-hoc analyses were used where appropriate. Alpha values of *p* < 0.05 were set for significance testing for all statistical analyses. Please see Supplementary Information (Table S9) for GLiM variable combinations analysed.

## Supplementary Information

Below is the link to the electronic supplementary material.

Supplementary Material 1

## Data Availability

The datasets analysed for this study are available from the corresponding author E.L.N. upon reasonable request.
