# Supplementary material for: The impact of the BDNF Val66Met genotype on intrusive memories following trauma exposure and in PTSD is moderated by sex and timing of trauma exposure
Source: Sci Rep. 2025 Aug 22;15:30863. doi: 10.1038/s41598-025-13812-8 (PMC12373918; doi:10.1038/s41598-025-13812-8)
Supplement: Supplementary file 1 — Supplementary Material 1 [file 41598_2025_13812_MOESM1_ESM.pdf]

Supplementary Information

The impact of the BDNF Val66Met genotype on intrusive memories and in post-traumatic stress disorder is moderated by sex and timing of trauma exposure

Emma Louise Nicholson[1*], Michael Garry[2], Luke J. Ney[3], Chia-Ming K. Hsu[2], Daniel V. Zuj[2,4] and Kim L. Felmingham[1]

[1]Melbourne School of Psychological Sciences, University of Melbourne, Australia

[2]School of Psychological Sciences, University of Tasmania, Australia

[3]School of Psychology and Counselling, Faculty of Health, Queensland University of Technology, Australia

[4]Experimental Psychopathology Lab, Department of Psychology, Swansea University, UK

[*]Corresponding author: Emma Nicholson, Melbourne School of Psychological Sciences, Redmond Barry Building, University of Melbourne Parkville VIC 3010 Australia

Email address: elnicholson@student.unimelb.edu.au

Phone number: +61405978831

Table S1

Significant Sidak Pairwise Comparisons With p-values and 95% CIs Following Up Significant ANOVAs for Demographic Clinical Measures Across Groups

			95% Confidence Intervals	
Measure	Groups compared	*p* value	Lower bound	Upper bound
Age	Controls and TE	<.001	-7.72	-2.39
	Controls and PTSD	<.001	-12.55	-3.94
BMI	PTSD and Controls	<.001	0.90	4.98
DASS				
- depression	PTSD and TE	< .001	3.18	6.84
	PTSD and controls	< .001	4.14	7.70
- anxiety	PTSD and TE	< .001	4.32	6.40
	PTSD and controls	< .001	4.87	7.00
- stress	PTSD and TE	< .001	5.00	8.41
	PTSD and controls	< .001	6.70	10.04
	TE and controls	.001	.57	2.76
PCL	PTSD and TE	< .001	24.80	32.47
	PTSD and controls	< .001	31.33	39.15
AUDIT	PTSD and TE	< .020	.34	4.69
	PTSD and controls	.004	.82	5.14

Table S2a.

Mean Number of Intrusive Memories reported across Group, Sex and Genotype

Group	Genotype	Sex	Mean	*SE*	*95%* Confidence Intervals	
					Lower bound	Upper Bound
Control	Val/Val	Male	.11	.06	.04	.32
		Female	.33	.12	.17	.67
	Val/Met	Male	.05	.05	.01	.35
		Female	.59	.18	.32	1.07
TE	Val/Val	Male	.62	.18	.35	1.09
		Female	1.00	.20	.68	1.48
	Val/Met	Male	.18	.09	.06	.49
		Female	.75	.18	.47	1.21
PTSD	Val/Val	Male	.88	.45	.32	2.39
		Female	1.73	.25	1.30	2.30
	Val/Met	Male	.87	.33	.42	1.82
		Female	1.27	.29	.81	1.99

Note: SE= standard error

Table S2b.

Mean Number of Intrusive Memories reported across Groups

Group	Mean	Standard Error	*n*
Control	.18	.06	105
TE	.54	.09	118
PTSD	1.14	.20	53

Table S2c.

Mean Number of Intrusive Memories reported across BDNF Genotype

Genotype	Mean	Standard Error	*n*
Val/Val	.57	.09	160
Val/Met	.41	.09	116

Table S2d.

Mean Number of Intrusive Memories reported across Sex

Sex	Mean	Standard Error	*n*
Male	.28	.07	108
Female	.83	.09	168

Table 3a.

Mean Number of Intrusive Memories Reported by Trauma Exposed Participants across Groups, Sex and Genotype

Group	Genotype	Sex	Mean	*SE*	*95%* Confidence Intervals	
					Lower bound	Upper Bound
TE	Val/Val	Male	.64	.18	.37	1.13
		Female	1.00	.20	.68	1.49
	Val/Met	Male	.08	.04	.03	.21
		Female	.78	.20	.47	1.31
PTSD	Val/Val	Male	.59	.312	.21	1.66
		Female	1.64	.22	1.25	2.15
	Val/Met	Male	.82	.23	.46	1.45
		Female	1.44	.28	.98	2.12

Note: SE= standard error

Table S3b.

Mean Number of Intrusive Memories Reported by Trauma Exposed Participants across Groups

Group	Mean	Standard Error	*n*
TE	.44	.08	112
PTSD	1.03	.16	53

Table S3c.

Mean Number of Intrusive Memories Reported by Trauma Exposed Participants across BDNF Genotype

Genotype	Mean	Standard Error	*n*
Val/Val	.89	.14	102
Val/Met	.51	.08	63

Table S3d.
Mean Number of Intrusive Memories Reported by Trauma Exposed Participants across Sex

Sex	Mean	Standard Error	*n*
Male	.39	.08	61
Female	1.17	.12	104

Table S3e.
Mean Number of Intrusive Memories Reported by Trauma Exposed Participants across Developmental Trauma Stage

Genotype	Mean	Standard Error	*n*
Child (<18yrs)	.73	.12	94
Adult (18 and above)	.63	1.00	71

Table S4a.
Mean Distress Levels Reported across Group, Sex and Genotype

Group	Genotype	Sex	Mean	*SE*	*95%* Confidence Intervals	
					Lower bound	Upper Bound
Control	Val/Val	Male	.33	.27	- .20	.87
		Female	.45	.20	.06	.85
	Val/Met	Male	.13	.33	-.53	.78
		Female	1.00	.25	.51	1.49
TE	Val/Val	Male	.72	.22	.29	1.16
		Female	.81	.17	.48	1.14
	Val/Met	Male	.10	.30	- .48	.68
		Female	.95	.21	.54	1.36
PTSD	Val/Val	Male	1.86	.36	1.16	2.55
		Female	1.92	.19	1.55	2.29
	Val/Met	Male	1.86	.36	1.16	2.55
		Female	1.55	.28	.99	2.10

Note: SE= standard error

Table S4b.
Mean Distress Levels Reported across Groups

Group	Mean	S.E.	*n*
Control	.48	.13	56
TE	.64	.12	79
PTSD	1.79	.15	50

Table S4c.
Mean Distress Levels Reported across BDNF Genotype

Genotype	Mean	SE	*n*
Val/Val	1.02	.10	115
Val/Met	.93	.12	70

Table S4d.
Mean Distress Levels Reported across Sex

Sex	Mean	SE	*n*
Male	.83	.13	62
Female	1.11	.09	123

Table S5a.

Mean Vividness of Intrusive Memories Reported across Group, Sex and Genotype

Group	Genotype	Sex	Mean	*SE*	*95%* Confidence Intervals	
					Lower bound	Upper Bound
Control	Val/Val	Male	1.58	.30	.21	1.60
		Female	.82	.26	.44	1.53
	Val/Met	Male	.50	.35	.13	2.00
		Female	1.54	.37	.96	2.47
TE	Val/Val	Male	1.35	.29	.89	2.05
		Female	1.42	.23	1.04	1.94
	Val/Met	Male	.70	.40	.23	2.15
		Female	1.25	.27	.82	1.92
PTSD	Val/Val	Male	2.14	.47	1.39	3.30
		Female	2.40	.21	2.02	2.85
	Val/Met	Male	1.86	.47	1.13	3.05
		Female	1.73	.32	1.21	2.48

Note: SE= standard error

Table S5b.

Mean Vividness of Intrusive Memories Reported across Groups

Group	Mean	S.E.	*n*
Control	.78	.19	55
TE	1.14	.19	78
PTSD	2.02	.20	50

Table S5c.

Mean Vividness of Intrusive Memories across BDNF Genotype

Genotype	Mean	SE	*n*
Val/Val	1.29	.15	114
Val/Met	1.14	.19	69

Table S5d.

Mean Vividness of Intrusive Memories across Sex

Sex	Mean	SE	*n*
Male	1.02	.19	61
Female	1.45	.13	122

Table S6a.

Mean Vividness of Intrusive Memories Reported in Participants Exposed to Trauma across Group, Sex and Genotype

Group	Genotype	Sex	Mean	*SE*	*95%* Confidence Intervals	
					Lower bound	Upper Bound
TE	Val/Val	Male	1.42	.32	.92	2.19
		Female	1.38	.21	1.02	1.87
	Val/Met	Male	.35	.16	.15	.84
		Female	1.31	.32	.81	2.12
PTSD	Val/Val	Male	2.07	.42	1.39	3.08
		Female	2.38	.22	1.98	2.85
	Val/Met	Male	1.49	.47	.81	2.76
		Female	1.99	.34	1.43	2.77

Table S6b.

Mean Vividness of Intrusive Memories Reported in Participants Exposed to Trauma across Groups

Group	Mean	S.E.	*n*
TE	.98	.15	74
PTSD	1.96	.20	50

Table S6c.

Mean Vividness of Intrusive Memories Reported in Participants Exposed to Trauma across BDNF Genotype

Genotype	Mean	SE	*n*
Val/Val	1.76	.15	79
Val/Met	1.08	.17	45

Table S6d.

Mean Vividness of Intrusive Memories Reported in Participants Exposed to Trauma across Sex

Sex	Mean	SE	*n*
Male	1.12	.171	40
Female	1.71	.150	84

Table S7.

Mean Number of Negative Images Correctly Recalled Reported across Group, Sex and Genotype

Group	Genotype	Sex	Mean	*SE*	*95%* Confidence Intervals	
					Lower bound	Upper Bound
Control	Val/Val	Male	5.26	.35	4.57	5.95
		Female	4.77	.30	4.18	5.36
	Val/Met	Male	4.21	.44	3.35	5.07
		Female	4.66	.37	3.93	5.38
TE	Val/Val	Male	4.21	.36	3.50	4.91
		Female	5.03	.29	4.45	5.60
	Val/Met	Male	4.94	.56	3.84	6.04
		Female	4.66	.32	4.02	5.28
PTSD	Val/Val	Male	5.13	.76	3.63	6.61
		Female	4.81	.37	4.08	5.53
	Val/Met	Male	4.13	.45	3.25	5.00
		Female	5.27	.53	4.23	6.31

Note: SE= standard error

Table S8.
Mean Number of Negative Images Correctly Recalled Reported across Group, Sex and Genotype in Trauma Exposed Participants

Group	Genotype	Sex	Mean	*SE*	*95%* Confidence Intervals	
					Lower bound	Upper Bound
TE	Val/Val	Male	4.18	.37	3.46	4.90
		Female	5.04	.30	4.45	5.63
	Val/Met	Male	4.91	.53	3.87	5.96
		Female	4.76	.38	4.02	5.51
PTSD	Val/Val	Male	5.12	.91	3.34	6.90
		Female	4.91	.36	4.21	5.61
	Val/Met	Male	4.21	.71	2.83	5.59
		Female	5.47	.64	4.22	6.71

Note: SE= standard error

Table S9.
Generalised Linear Model Variable Combinations Analysed

Model	Response Variable	Predictor Variables	Co-variates
Generalised Linear Model (GLiM)	Number of Intrusive Memories	Group<br>Val66Met Genotype<br>Sex	Ethnicity<br>BMI
GLiM	Number of Intrusive Memories	Group<br>Val66Met Genotype<br>Sex	Ethnicity<br>BMI<br>Stress
GLiM	Number of Intrusive Memories	Group<br>Val66Met Genotype<br>Sex	Ethnicity<br>BMI<br>Age
GLiM	Number of Intrusive Memories	Group<br>Val66Met Genotype<br>Sex	Ethnicity<br>BMI<br>AUDIT scores
GLiM	Number of Intrusive Memories	Group (TE & PTSD only)<br>Val66Met Genotype<br>Sex<br>Child/Adult trauma	Ethnicity<br>BMI
GLiM	Number of Intrusive Memories	Group (TE & PTSD only)<br>Val66Met Genotype<br>Sex	Ethnicity<br>BMI<br>Number of traumas experienced
GLiM	Level of Distress	Group<br>ValMet Genotype<br>Sex	Ethnicity<br>BMI
GLiM	Level of Distress	Group (TE & PTSD only)<br>ValMet Genotype<br>Sex<br>Child/Adult trauma	Ethnicity<br>BMI

GLiM	Level of Distress	Group (TE & PTSD only)<br>ValMet Genotype<br>Sex	Ethnicity<br>BMI<br>Number of traumas experienced
GLiM	Vividness of Intrusive Memories	Group<br>ValMet Genotype<br>Sex	Ethnicity<br>BMI
GLiM	Vividness of Intrusive Memories	Group (TE & PTSD only)<br>ValMet Genotype<br>Sex<br>Child/Adult trauma	Ethnicity<br>BMI
GLiM	Vividness of Intrusive Memories	Group (TE & PTSD only)<br>ValMet Genotype<br>Sex	Ethnicity<br>BMI<br>Number of traumas experienced
GLiM	Number of Negative Images Correctly Recalled	Group<br>ValMet Genotype<br>Sex	Ethnicity<br>BMI
GLiM	Number of Negative Images Correctly Recalled	Group (TE & PTSD only)<br>ValMet Genotype<br>Sex<br>Child/Adult trauma	Ethnicity<br>BMI
GLiM	Number of Negative Images Correctly Recalled	Group (TE & PTSD only)<br>ValMet Genotype<br>Sex	Ethnicity<br>BMI<br>Number of traumas experienced

Note: As per the initial models with Intrusive Memories as the dependent variable (see above in red text), all subsequent models included separate analyses with stress, age, and audit scores added individually as covariates in addition to ethnicity, BMI and number of traumas experienced.
